# Infectious Aerosol Capture Mask as Environmental Control to Reduce Spread of Respiratory Viral Particles

**DOI:** 10.3390/v14061275

**Published:** 2022-06-11

**Authors:** Joshua L. Santarpia, Nicholas W. Markin, Vicki L. Herrera, Daniel N. Ackerman, Danielle N. Rivera, Gabriel A. Lucero, Steven J. Lisco

**Affiliations:** 1 Department of Pathology and Microbiology, University of Nebraska Medical Center, Omaha, NE 68198, USA; vlherrera@unmc.edu; 2 The Global Center for Health Security, University of Nebraska Medical Center, Omaha, NE 68198, USA; danielle.rivera@unmc.edu; 3 The National Strategic Research Institute, Omaha, NE 68106, USA; dackerman@nsri.nebraskaresearch.gov (D.N.A.); glucero@nsri.nebraskaresearch.gov (G.A.L.); 4 Department of Anesthesiology, University of Nebraska Medical Center, Omaha, NE 68198, USA; nmarkin@unmc.edu (N.W.M.); steven.lisco@unmc.edu (S.J.L.)

**Keywords:** airborne isolation, aerosol capture, SARS-CoV-2

## Abstract

Negative pressure isolation of COVID-19 patients is critical to limiting the nosocomial transmission of SARS-CoV-2; however, airborne isolation rooms are limited. Alternatives to traditional isolation procedures are needed. The evaluation of an Infectious Aerosol Capture Mask (IACM) that is designed to augment the respiratory isolation of COVID-19 patients is described. Efficacy in capturing exhaled breath aerosols was evaluated using laboratory experimentation, computational fluid dynamics (CFD) and measurements of exhaled breath from COVID-19 patients and their surroundings. Laboratory aerosol experiments indicated that the mask captured at least 99% of particles. Simulations of breathing and speaking showed that all particles between 0.1 and 20 µm were captured either on the surface of the mask or in the filter. During coughing, no more than 13% of the smallest particles escaped the mask, while the remaining particles collected on the surfaces or filter. The total exhaled virus concentrations of COVID-positive patients showed a range from undetectable to 1.1 × 10^6^ RNA copies/h of SARS-CoV-2, and no SARS-CoV-2 aerosol was detected in the samples collected that were adjacent to the patient when the mask was being worn. These data indicate that the IACM is useful for containing the exhaled aerosol of infected individuals and can be used to quantify the viral aerosol production rates during respiratory activities.

## 1. Introduction

In 2019, a novel coronavirus was isolated from a series of patients in Wuhan, China [1]. The virus, SARS-CoV-2, and its clinical presentation, coronavirus disease 2019 (COVID-19) has resulted in a pandemic with significant morbidity and mortality worldwide. As part of the effort to understand the disease and its transmission, several studies have demonstrated that SARS-CoV-2 can be transmitted in contact, droplet, and aerosolized forms [2,3]. These studies showed that viral RNA and viable virus was seen in the surface samples that were taken from hospital room surfaces in COVID-19 units [4,5]; that the virus can survive on surfaces for prolonged periods of time; and that samples that were taken from the air and from surfaces 5+ m from patients were also contaminated, thus strengthening the belief that aerosol transmission is one such route of transmission [6,7].

To minimize the nosocomial transmission of SARS-CoV-2, airborne precautions are critical in the hospital setting. The need to protect against the transmission of SARS-CoV-2 has resulted in the implementation of several precautions to reduce spread, including the use of N-95 respirators, single-room isolation and negative-pressure isolation rooms, or airborne infection isolation rooms (AIIRs) [8]. The concern for airborne transmission has been evaluated by multiple groups with mixed results. Although a few reports suggest that no SARS-CoV-2 RNA was isolated from air samples, factors such as high room ventilation, variability in respiratory aerosol production, or under sampling of the environment may account for these findings. It is possible that the absence of findings is not representative [9,10]. Aerosolized virus has been detected in the hospital setting by several independent groups. Liu et al. identified viral RNA in small particles with aerodynamic diameters ranging from 0.25 to 2.5 µm in both direct patient care areas and staff areas [11]. Song et al. identified low levels of viral RNA sampled from AIIR air and detected on multiple surfaces [12]. Santarpia et al. demonstrated evidence for both findings, showing the significant airborne contamination of air samples that were taken at 2 m from the patient [13] and virus from aerosolized particles <1 µm that replicated in cell culture [14].

Aerosols are generated naturally from various sites within the respiratory tree with larger droplet and droplet nuclei arising from more proximal locations, such as the mouth (100 µm), medium sized particles from the larynx (10 µm) and smaller particles from the distal airways (1 µm) [15,16]. Therefore, depending on the timing of COVID-19 infection, it is possible to distribute the virus in various particle sizes with the smallest particle sizes theoretically carrying infectious particles if the distal airway fluid contains virions. Once these small particles are aerosolized, they can remain suspended and be distributed throughout a room based on air current and fresh air exchanges [17].

Recommendations for patient care in the setting of COVID-19 include infection control recommendations and engineering controls, such as placing patients in single-occupancy AIIRs, routine decontamination, and the use of face masks to reduce the risk of spread to health care workers (HCW) [18,19,20]. For many hospitals and healthcare systems, it has been a challenge to find enough negative-pressure AIIRs to appropriately triage and safely manage patients with COVID-19 during the pandemic. Using surgical masks to mitigate the aerosol that is produced by infected patients has shown to be effective, particularly for particles that are larger than 5 µm [21,22]. However, these same studies indicate less efficacy in reducing aerosols that are smaller than 5 µm, so surgical masks alone cannot replace AIIRs in minimizing the risk of the nosocomial transmission of SARS-CoV-2. To address this need, our team developed a face-fitting mask called the infectious aerosol capture mask (IACM) from preexisting components for the purpose of generating a personal respiratory-isolation environment. The mask serves as an engineering control to reduce the risk of both droplet nuclei and aerosolized particles from entering the environment. The IACM functions by creating a negative pressure space adjacent to the patients’ face using hospital wall suction. The suction draws air from the environment along with the exhaled gases and particles that the patient produces while wearing the IACM. The exhaled particles and gasses pass through the mask into a viral filter and then, via an adapter, the airflow is carried out through the hospital vacuum line. Herein we describe our findings from a concurrent collection of exhaled gases from patients with confirmed positive reverse transcription polymerase chain reaction (RT-PCR+) SARS-CoV-2 infection and symptoms of COVID-19 during simultaneous environmental air sampling from 2 m from the individual wearing the IACM.

## 2. Materials and Methods

**Figure 1:**
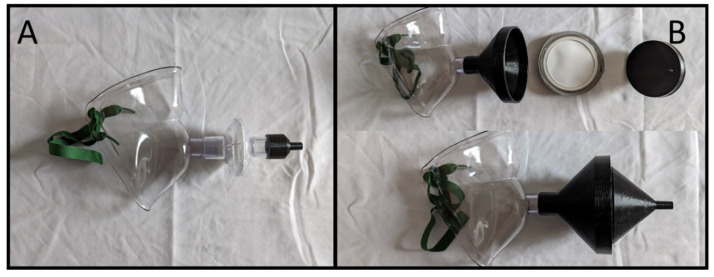
Images and designs of IACM components. (**A**) Assembled IAMC device using a viral filter. Suction can be attached at the end of the custom adaptor (right). (**B**) IACM device used to collect respiratory particles for analysis. This version used a water-soluble 80 mm gelatin filter to collect particles for easy recovery. In addition, sterile gauze was used to swab the interior surface of the clear mask to recover deposited particulate. Detailed designs of the custom filter housing used to attach 80 mm filter to mask and suction, and the custom adaptor used to connect viral filter to suction tubing are available in the Supplementary Materials (Figures S1 and S2).

The IACM (Figure 1) was fashioned from commercially available medical equipment and a single 3D printed adapter. The face fitting portion is a Hudson RCI aerosol face tent (Teleflex, Morrisville, NC, USA) to which a Hudson RCI Viral Filter (Teleflex) is connected via the 22 mm connection (Figure 1A). The viral filter is then connected via a 3D printed adapter (Figure 1B) with a 22 mm ID connection on one side and a 9 mm OD connection on the other side via suction tubing to either the hospital vacuum line or a portable vacuum pump. The vacuum is set to a minimum of −100 torr to generate approximately 28.3 Lpm of airflow. During testing in the patient care setting for this effort, the Hudson RCI Viral Filter was replaced with an 80 mm gelatin filter (Sartorius, Gmbh, Goettingen, Germany) using a 3D printed holder. Use of the gelatin filter allowed the easy assay of material that was collected by the mask.

**Figure 2:**
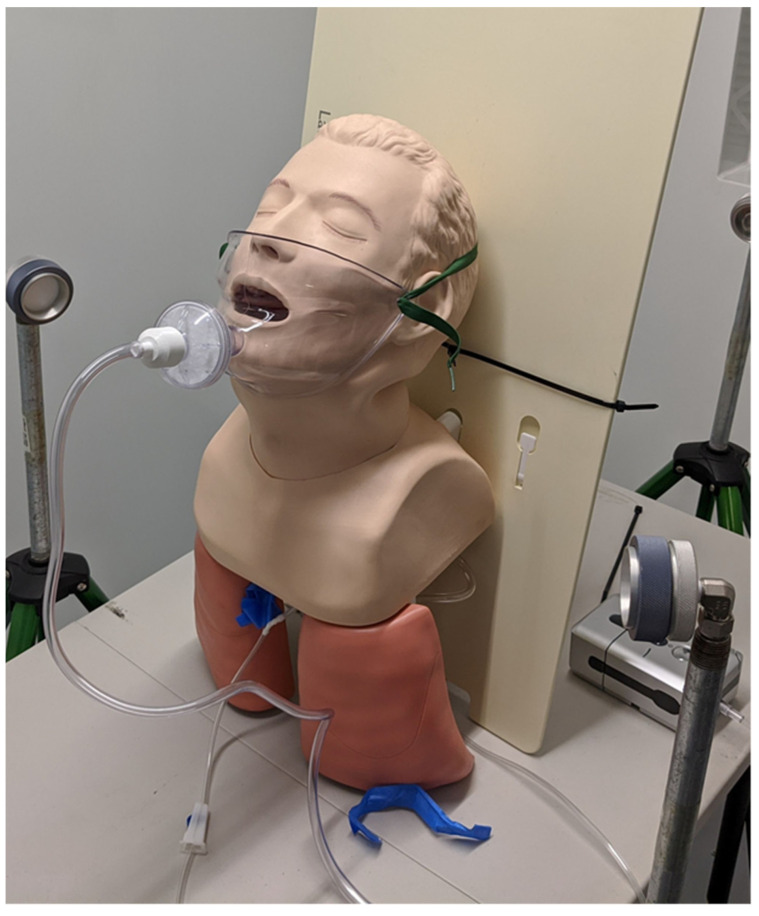
Photograph of the experimental arrangement used in preliminary evaluation of the IACM. A schematic drawing is available in the Supplementary Materials (Figure S3).

After initial development, preliminary experiments were performed to evaluate the efficacy of IACM at capturing aerosol particles, and to evaluate the impact of reducing the airflow rate on efficacy. This experiment was performed in a HEPA-filtered aerosol chamber with 1 um DNA coated polystyrene latex (PSL) spheres (see Kinahan et al. [23]). Beads were aerosolized via the airway of a mannequin at approximately 6 Lpm using a DeVilbiss Traveler Nebulizer and a Hudson Micro Mist medical nebulizer, into an approximately 12 m^3^ HEPA-filtered chamber in stagnant air (no air changes). Air near the mannequin was sampled at 50 Lpm for 15 min during aerosol generation and was collected using 47 mm glass-fiber filters that were housed in BGI open-face filter holders (Mesa Labs, Lakewood, CO, USA; Figure 2). The filters were set at a height that matched that of the mannequin and approximately 30 cm from the mouth of the mannequin. Between experiments, the air in the chamber was exhausted for at least 100 air changes to ensure no cross-contamination. Two airflow conditions were studied: 28.3 Lpm through the mask and 14.2 Lpm through the mask, and these were generated using −100 torr and −50 torr, respectively. The filters were collected and transferred from the filter housing to a 50 mL conical tube and rehydrated in 10 mL of filtered DI water, then diluted to 1:10 in filtered DI water.

To quantify the number of beads that were collected in each sample, all samples were processed in triplicate, using a Bio-Rad CFX Connect 96-well system (BioRad Laboratories, Hercules, CA, USA) with no-template controls and representative samples of the bead stock as a positive control. The reactions used 5 µL of unprocessed sample. The cycle times were compared with the standard curve of the original bead stock to determine the concentration of beads in each sample (Table 1). The concentration of beads that were sampled in the air and on each surface was then determined using the sampling and recovery parameters described above.

**Figure 3:**
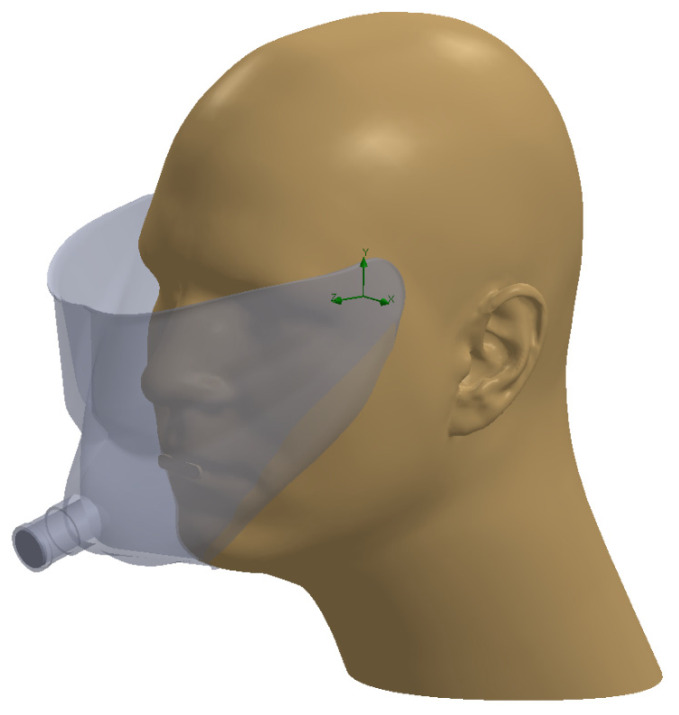
Solid model used for computational fluid dynamics simulations. Airflow is from the opening of the mask at the top through the dark grey outlet of the mask. Particle trajectories are initialized at the opening of the mouth.

To better describe and understand the performance of the IACM we performed simple computational fluid dynamics simulations using COSMOS FloWorks (SolidWorks, Waltham, MA, USA), which utilizes a finite volume method to solve the fluid dynamics equations, and Lagrangian particle tracking to determine the particle position. The model is set up so that the mask is sealed to the face, except at the top (Figure 3). A mask flow of 28.3 Lpm was modelled entering from the gap between the face and the mask and exiting through the port for the filter, which corresponded to the volume of air passing through the mask at −100 torr. Flow simulations were initialized using multiple particle sizes (0.1, 0.5, 1, 5, 10 and 20 µm) and 3 flow conditions. Initial particle velocities of 1, 4 and 15 m/s were taken from Tang et al. [24] and Kwon et al. [25] to represent mouth breathing, talking, and coughing, respectively. The particles were modelled as spheres with the density of water. If particles pass through the exit plane of the mask, they are considered filtered. If particles impact any surface, including the mask or face, they will be absorbed and not re-entrained into the flow. One hundred individual trajectories for each particle size were initialized at regular spacings across the mouth area of the model framework (marked by the oval shaped area in Figure 3).

Environmental samples were collected concurrently in 8 patients that were known to be RT-PCR SARS-CoV-2 positive with an admitting diagnosis of respiratory insufficiency due to COVID-19. The IACM worn by patients utilized a modified filter holder that was designed specifically for 80 mm gelatin filters. At the time of sample collection, inpatients were in AIIRs in the COVID unit of the hospital. Investigators utilized PPE that was appropriate for airborne infection precautions. As exhaled gases are not considered to be human biological material and no identifiers were recorded, the collection was considered not human subject research. Patients did provide verbal consent to wearing the IACM following an explanation of its function and purpose. Patients wore the IACM for 30 min during which air samples were collected immediately adjacent to the bed or chair where the individual wearing the IACM was located, generally within 1 m. During the collection period, the IACM was connected to the hospital vacuum line or a vacuum pump drawing at least 28.3 Lpm at the start of collection, with the suction regulator set to a minimum of −100 torr. Unregulated wall suction was utilized if a regulator was not present or available at bedside.

The collected environmental samples were performed using a Sartorius MD8 Airport that was set to collect 1000 L of air at 30 Lpm. A swab, premoistened with 3 mL of sterile PBS was used to sample the interior surface of the mask; the surface sample from the IACM and the gelatin filter were both collected and analyzed for SARS-CoV-2. Samples were also collected with the Sartorius MD8 from the rooms of 6 other patients that were known to be RT-PCR SARS-CoV-2 positive with admission for COVID-19, using the same method, to serve as a control for environmental samples in the absence of the IACM.

Swab and filter samples were recovered following the methods of Santarpia et al. [13]. In brief, the swab samples were recovered by adding 15 mL of sterile PBS and manually shaking the conical tube for 1 min. The 80 mm gelatin filters were dissolved in 15 mL of sterile PBS. RNA extractions were performed using a Qiagen DSP Virus Spin Kit (QIAGEN GMbH, Hilden, Germany). Each PCR run included a positive control using isolated viral RNA and a negative, no template control of nuclease-free water. The reactions were set up and run with initial conditions of 10 min at 55 °C and 4 min at 94 °C, then 45 cycles of 94 °C for 15 s and 58 °C for 30 s, QuantStudio™ 3 (Applied Biosytems™, Inc., Waltham, MA, USA).

To quantify the virus present in each sample from the measured Ct values that were obtained from the real time RT-PCR (rRT-PCR), a standard curve was developed using RNA that was extracted from a known quantity of SARS-CoV-2 virus (BEI_USA-WA1/2020), cultivated in Vero E6 cells (as described in Santarpia et al. [14]). Two lots of assays were used in this study. Both had identical sequences but were obtained from IDT (Integrated DNA Technologies, Coralville, IA, USA) at different times. Standard curves were run in triplicate beginning at a concentration of 1.3 × 10^2^ pfu/mL, and as determined by plaque assay. The data were fit with the following exponential functions:

(1) $$Equivalent\;Viral\;Titer\;\left(\frac{\mathrm{pfu}}{\mathrm{mL}}\right)=6.0\times 10^{9}\cdot e^{-0.707*C_{t}}\;R^{2}=0.9557$$

(2) $$Equivalent\;Viral\;Titer\;\left(\frac{\mathrm{pfu}}{\mathrm{mL}}\right)=2.0\times 10^{8}\cdot e^{-0.689*C_{t}}\;R^{2}=0.9994$$

where Equation (1) applies to the environmental samples that were collected from patients not wearing the IACM, and Equation (2) applies to the environmental samples from patients wearing an IACM. Since the two assay lots had different responses, and many rRT-PCR runs were needed to analyze all samples in this study, several measures were taken to ensure intercomparability between all data. First, the same cycle threshold was used for all rRT-PCR sample runs with each assay lot. Second, standard curves were developed for each assay lot with the same lot of viral RNA. The derived exponential functions (above) were then used to convert the measured *Ct* values to an equivalent viral titer (e-*pfu*) for each sample, prior to comparison. In this way, the *Ct* value from each sample is converted into an equivalent viral titer based on the same extracted RNA, which more accurately represents the RNA that can be recovered from the virus through the extraction and reverse-transcriptase processes, which are not accurately captured using a synthetic DNA control. It is important to note that e-*pfu* is not the same as the number of RNA copies nor the number of virions that may be present in a clinical sample. Therefore, the conversion factor developed in Santarpia et al. [14] of 1.35 × 10^6^ +/− 6.29 × 10^5^ RNA copies/e-*pfu*, based on the same gene target, is used to convert the e-*pfu* to copies for easier comparison with other studies.

## 3. Results

Preliminary testing with DNA-coated 1 µm PSL beads indicated that at either 14.2 or 28.3 Lpm, more than 99% of the PSL beads were captured by the mask, when compared to no flow through the mask (Table 2). The concentrations that were measured during the tests with either 14.2 or 28.3 Lpm were not significantly different (*p* = 0.37), indicating that differences in the calculated reductions are also not significant. Kinahan et al. [23] showed that in similar chamber studies, surgical masks only reduced the aerosol that was produced by the mannequin by 7.6%. It is important to note that since the flow rate of the nebulized particles may be much lower than the actual exhalation rate of a wearer, and since the flow was constant rather than following a normal, sinusoidal breathing pattern, the reductions may not be indicative of particle reductions when worn by a patient.

The preliminary findings from the chamber tests indicated that further study of the IACM was warranted. COSMOS FloWorks was used to estimate the efficacy of the mask over a range of particle sizes and respiratory activities (breathing, speaking, and coughing). In most scenarios modelled, the total capture of aerosols that were produced by a wearer was greater than 87% (Table 3 and Table S1). The only modelled scenarios where less than 100% of the particles were either filtered or impacted on modelled surfaces were during coughing. In the case of coughing, a larger fraction of particles of 1 µm or smaller were lost when compared to the larger particles (Table 3 and Table S1). The modelled results also indicate that the final location of a particle (filter, mask/face surface, or escaped) is more dependent on the initial velocity of the particle (assumed based on respiratory activity) than on particle size. Therefore, no assumption about particle size can be easily made based solely on the location where particles are found, since a person wearing a mask may generate particles by any respiratory activity.

When the IACM was worn by hospitalized patients, no SARS-CoV-2 aerosol particles were sampled in the room near them (Table 4, first 8 rows). Further, analysis of the filter and swabs on the inside of the mask indicate the presence of SARS-CoV-2 in six of the eight patients, confirming that respiratory particulate containing the virus was produced by those patients during the time that they wore the mask. This can be compared to similar measurements made of other patients who were not wearing the IACM but were known to the RT-PCR+ for SARS-CoV-2 (Table 4, last 4 rows), where aerosols were observed, and where RNA was recovered from the air that was sampled during such conditions.

## 4. Discussion

These findings demonstrate that the IACM is effective at capturing respiratory particulate that is produced by the wearer and may be additive to currently used respiratory isolation strategies. This has been demonstrated through complementary simulations and measurements around hospitalized patients. Initial simulation experiments with inert particles demonstrated the effective flow rate range for containing expelled particles. These findings were extended to multiple particle sizes and initial velocities that were representative of human breathing, speaking, and coughing using computational modeling. Finally, a practical demonstration showed no evidence of SARS-CoV-2 aerosol sampled from room air near the patients, while simultaneously demonstrating high levels of viral RNA on the mask and filter components of the IACM. These real-world collections occurred in patients that were hospitalized for symptomatic COVID-19, receiving oxygen per nasal cannula, and speaking and coughing during the collection period.

The routine use of surgical masks in non-AIIR situations may be less than ideal, especially if transmission by aerosol is expected. Demonstrations in prior studies show a less than 10% reduction in aerosolized 1 µm particles [23], which are on the upper bound of particles that are shown to contain culturable virus [13,14,26]. Therefore, while the public use of masks may reduce transmission, especially from larger particles (>5 µm) [21,22], the use of surgical masks as a source control in the healthcare setting around SARS-CoV-2 has limited value. The application of the IACM with its very high capture rate of both larger droplet and smaller aerosolized particles would be helpful in non-AIIR situations, specifically compared against the performance of a standard surgical mask.

Barriers that further reduce the presence and quantity of infectious particles across a broad range of particle size, such as the IACM could assist in minimizing contamination of the hospital environment. The IACM device may be particularly useful in areas of the hospital where patients who may be infected with SARS-CoV-2 cannot be effectively isolated by using typical means (AIIRs, etc.). This might include surgical recovery rooms, emergency departments, and any area of the hospital where air flow conditions are not suitable for isolation. Additionally, the IACM could be used in more challenging conditions, for example, mass triage situations, emergency patient transport, and locations with constrained resources, as the device would continue to perform as described as long as an adequate vacuum source, such as a portable suction device, is available.

Use of this device could also facilitate the cohorting of patients based on needs other than COVID-19, allowing improved care for patients with more specialized conditions (e.g., cardiology, etc.). An ongoing struggle with the COVID-19 pandemic is that patients requiring hospitalization for reasons other than COVID-19 infection are incidentally also infected with SARS-CoV-2. These concurrent infections further stress health care because they require patients who are admitted for a non-SARS-CoV-2 cause to be sequestered into a COVID-19 ward, which may negatively impact the access of the nursing and physician specialty teams. This device could extend space capabilities while maintaining provider safety and the safety of other patients. Due to its simplicity, it can be implemented in both metropolitan and rural hospitals, as well as the prehospital environment.

The device in its current form has limitations. The requirement for suction limits the movement of the patient, therefore it must be removed to facilitate eating and drinking, as well as moving around the room. As described here, it is most appropriate for patient wear while sedentary or resting. Furthermore, if the IACM is misadjusted in a way that significantly changes the airflow around the wearer’s face, it may limit its ability to collect exhaled particles. Additionally, over time the filter may load and cause reductions in flow that would limit the effectiveness of the device. During the short duration of these studies, filter loading did not appreciably impact the flow rate; however, this could impact the function of the device during extended wear, necessitating filter replacement. While there are limitations to the wearability of the version of the IACM that are described here, it does provide much better particle removal than most common masking options and does not restrict the breathing of the wearer.

The omicron-variant wave of the COVID-19 pandemic has seen further disruption to health care, not only because of the highly infectious nature of the variant [27], but as infections increase, there is an overall increase in the absolute numbers of patients admitted with COVID-19, as well as a high prevalence of patients admitted that are found to incidentally have RT-PCR+ SARS-CoV-2 infections. A recent report from New York state indicated that over 40% of patients that were admitted for non-COVID reasons were found to have RT-PCR+ SARS-CoV-2 results [28]. This is further complicated by the reality that the omicron variant is capable of infecting both individuals that have immunity from prior SARS-CoV-2 infections and those who received vaccination, with or without booster vaccination [29].

In addition to its clinical value, the IACM device also allows the measurement of the expired particulate from the people who wear it. The data from Table 4 indicate that the viral emission rates that were observed in hospitalized patients are similar to those that were reported in Ma, et al. [30] (ranging from no observation to ~10^6^ RNA copies per hour), but have a higher positivity rate (75% in this study compared to 26.9% in Ma et al.). To capture particles in the Ma study, the subjects were required to breath into the device. However, the IACM allows the capture of respiratory particulate during all respiratory activities. It is possible that the higher positivity rate is due to IACM’s ability to capture aerosol that is produced in activities other than breathing, but this could be a result of sampling bias due to the lower number of subjects (N = 8 compared to N = 52).

In conclusion, these data suggest that the IACM may be an additional environmental control and secondary alternative to standard AIIR conditions for those that are admitted with COVID-19. We cautiously suggest that it may also be beneficial in preliminarily isolating patients that may be infectious in triage situations or other environments with limited infrastructure. The IACM demonstrates a near complete capture of aerosolized particles, and because it is designed to have a gap at the top to allow entrainment of room air, it also allows the delivery of oxygen by nasal cannula without interrupting the function of the device. In fact, three of the patients that wore the mask during the study received oxygen while wearing the mask and still showed no evidence of aerosol in the room (Table 4). It is likely that there would be some conditions in which the IACM would not perform as well as it does in experimental conditions, such as if the lower portion is not well opposed to the face, permitting entrainment of air in areas that do not help to contain exhaled particles. Very high flow oxygen or vigorous coughing would likely result in the escape of some particles, but the overall performance of the IACM suggests that it may be helpful as an additional environmental control in various situations where standard negative-pressure isolation or other forms of AIIR cannot be accomplished. The individual isolation of infectious patients, which is possible with the IACM device, has the potential to improve healthcare delivery and safety in hospital and prehospital environments by providing an alternative to traditional patient isolation techniques.

## Figures and Tables

**Table 1:** Properties of DNA oligo attached to PSL beads.

Oligo Sequence	Forward Primer	Reverse Primer	Probe	Exponential Fit	R^2^
ttgttaaacctgtgaccacctgctaatcgtgcaaccttaccattcaggccgtgcgccgagcttacatgggcaattcaagtgtttgaggctcgggggcagg	CCT GTG ACC ACC TGC TAA TC	CCG AGC CTC AAA CAC TTG AA	TG CAA CCT T A CCA TTC AGG CCG T	Beads/mL = 9 × 10^8^ e^−0.649(Ct)^	0.9998

**Table 2:** Results of preliminary evaluation of IACM with DNA-tagged PSL beads.

	Sample	Mean Ct	Mean Concentration (beads/L of Air)	Stand. Dev.	Reuction Compared to No Flow
28.3 Lpm	Run 1 Sample 1	32.43	0.14	0.03	0.999
	Run 1 Sample 2	33.38	0.09	0.04	
	Run 2 Sample 1	32.56	0.13	0.02	
	Run 2 Sample 2	30.89	0.30	0.09	
14.2 Lpm	Run 1 Sample 1	33.32	0.09	0.00	0.998
	Run 1 Sample 2	34.28	0.06	0.00	
	Run 2 Sample 1	31.00	0.41	0.46	
	Run 2 Sample 2	30.97	0.43	0.50	
0 Lpm	Run 1 Sample 1	18.71	101.13	14.46	
	Run 1 Sample 1	19.20	79.44	3.07	
	Run 1 Sample 2	18.39	117.72	14.09	
	Run 2 Sample 1	17.12	215.44	3.92	

**Table 3:** Results of CFD modeling. Collection efficiencies are based on simulations using 100 particle releases.

Particles Collected on Filter						
Simulated Activity	0.1 µm	0.5 µm	1 µm	5 µm	10 µm	20 µm
Mouth Breathing	100%	100%	100%	100%	100%	99%
Speaking	32%	29%	29%	29%	25%	16%
Coughing	12%	11%	11%	12%	14%	8%
Particles Collected on Mask or Face						
Mouth Breathing	0%	0%	0%	0%	0%	1%
Speaking	68%	71%	71%	71%	75%	84%
Coughing	75%	82%	81%	84%	84%	91%
Particles Escaped						
Mouth Breathing	0%	0%	0%	0%	0%	0%
Speaking	0%	0%	0%	0%	0%	0%
Coughing	13%	7%	8%	4%	2%	1%

**Table 4:** Results of samples collected around hospitalized patients. The first 8 patients listed wore the device during collection, while the last 6 did not wear the device. ND indicates that target RNA was not detected. NA indicates that the experiment was not performed. “Failed” was used to indicate an experiment that was carried out, but where the sampler failed to operate correctly during the experiment.

Time Since First Reported Illness	Time Since First Reported Illness	Reported Symptoms	Talking/Coughing	Air Sample Room ePFU/L of Air in 1000 L Collected	Air Sample Room copies/L of Air in 1000 L Collected	Mask Filter ePFU/h	Mask Filter copies/h	Mask Swab ePFU/h	Mask Swab copies/h
5435	~14 days	Respiratory	no O_2,_ Limited talking, Coughing	ND	ND	3.11 × 10^−2^	4.21 × 10^4^	6.57 × 10^−3^	8.87 × 10^3^
5425	~5 days	GI, taste	no O_2,_ Talking no cough	ND	ND	ND	ND	ND	ND
5436	~7 days	Respiratory	no O_2,_ Talking no cough	ND	ND	ND	ND	1.29×10^−2^	1.74 × 10^4^
5444	~12 h	Respiratory	no O_2,_ Talking, 1 cough	ND	ND	1.68 × 10^−1^	2.27 × 10^5^	ND	ND
5436	~24 h	Respiratory	2 L O_2_, Talking, 33 coughs	Failed	Failed	1.36 × 10^−2^	1.84 × 10^4^	ND	ND
5450	~24 h	Respiratory	6 L O_2_, little talking	ND	ND	ND	ND	ND	ND
7442	~24 h	Respiratory	9 L O_2_, 10 coughs	ND	ND	ND	ND	3.37 × 10^−3^	4.55 × 10^3^
7480	~24 h	Respiratory	5 L O_2_, 3 coughs	ND	ND	2.48 × 10^−2^	3.35 × 10^4^	7.96 × 10^−1^	1.07 × 10^6^
7468	15 days	Respiratory	no O_2_, talking coughing	1.31 × 10^−3^	8.51 × 10	NA	NA	NA	NA
7472	10 days	Respiratory	no O_2_, talking, no coughing	5.09 × 10^−4^	3.30 × 10	NA	NA	NA	NA
5437	4 days	Respiratory	no O_2_, talking, coughing	1.34 × 10^−3^	8.67 × 10	NA	NA	NA	NA
5450	3 days	Respiratory	no O_2_, talking, coughing	1.27 × 10^−3^	8.25 × 10	NA	NA	NA	NA
5425	3 days (2 patients)	Respiratory	no O_2_, no talking, coughing	2.05 × 10^−3^	1.33 × 10^2^	NA	NA	NA	NA
5436	7 days	Respiratory	no O_2_, no talking, coughing	6.72 × 10^−4^	4.36 × 10	NA	NA	NA	NA

## Data Availability

All data has been included in the manuscript or in supplementary files.

## Supplementary Materials

The following supporting information can be downloaded at: https://www.mdpi.com/article/10.3390/v14061275/s1, Figure S1: Suction Adapter, Figure S2: Filter Adapter for Gelatin Filter. Figure S3: Aerosol Chamber Diagram. Table S1: Modelled Particle Data.
