# Supplementary material for: Infectious Aerosol Capture Mask as Environmental Control to Reduce Spread of Respiratory Viral Particles
Source: Viruses. 2022 Jun 11;14(6):1275. doi: 10.3390/v14061275 (PMC9227466; doi:10.3390/v14061275)
Supplement: Supplementary file 1 [file viruses-14-01275-s001.zip › Figure S2.pdf]

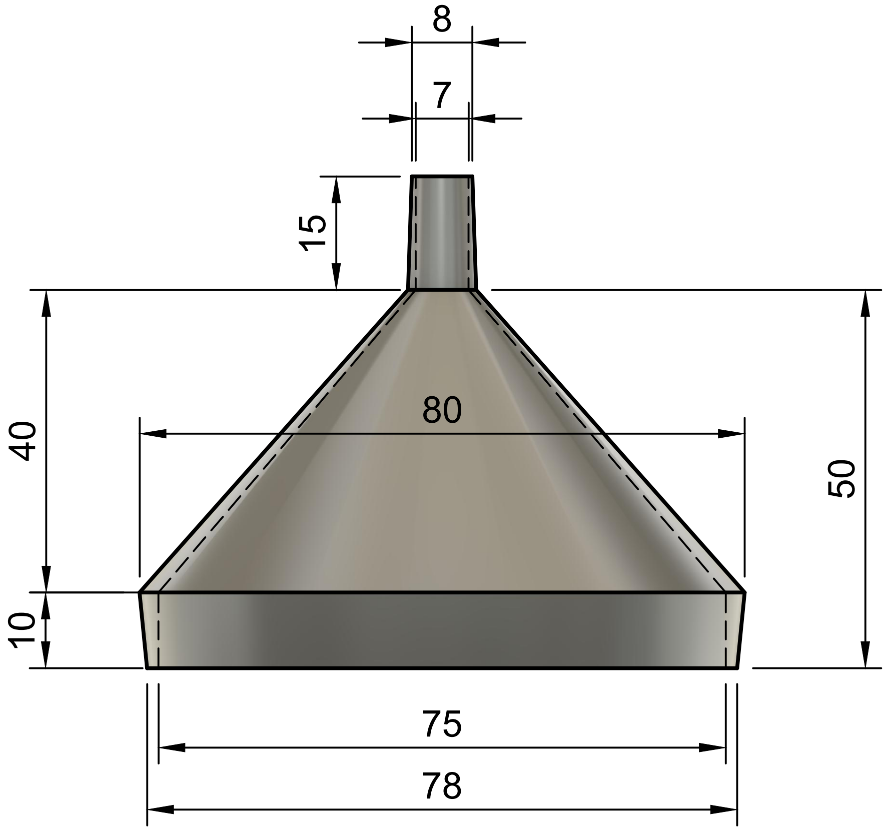

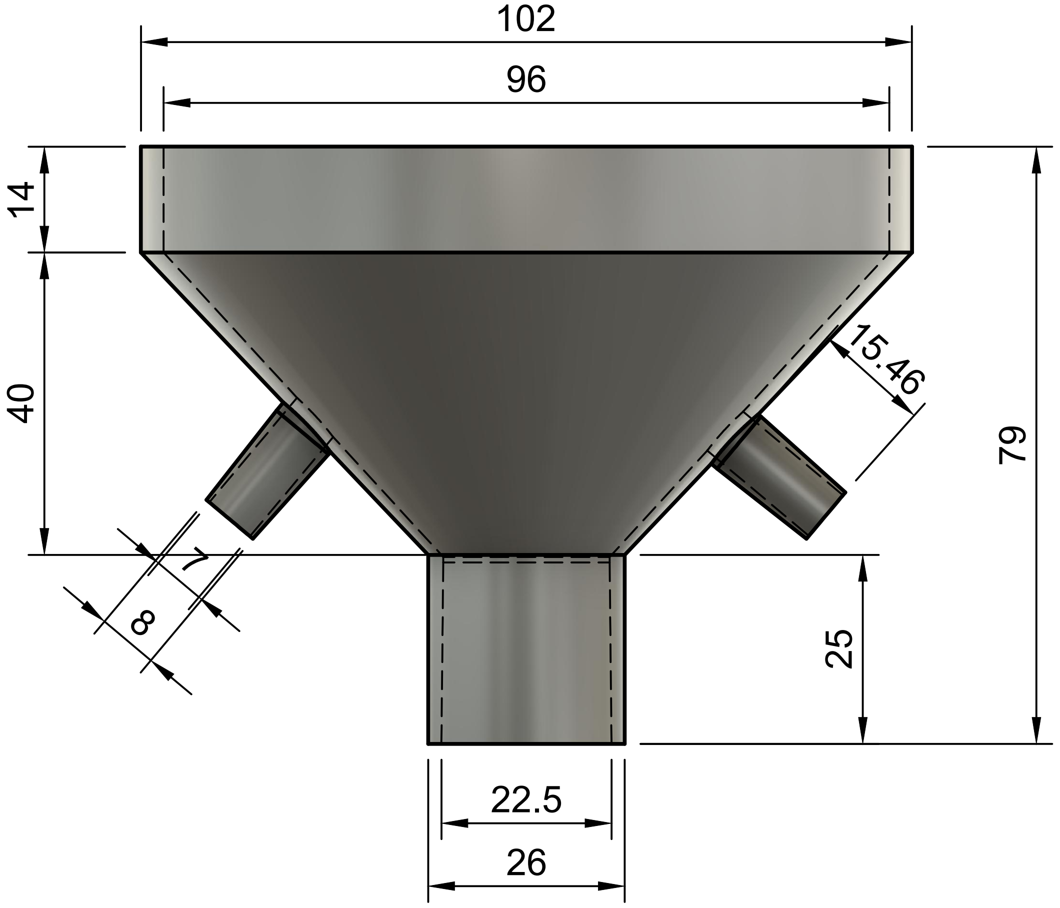

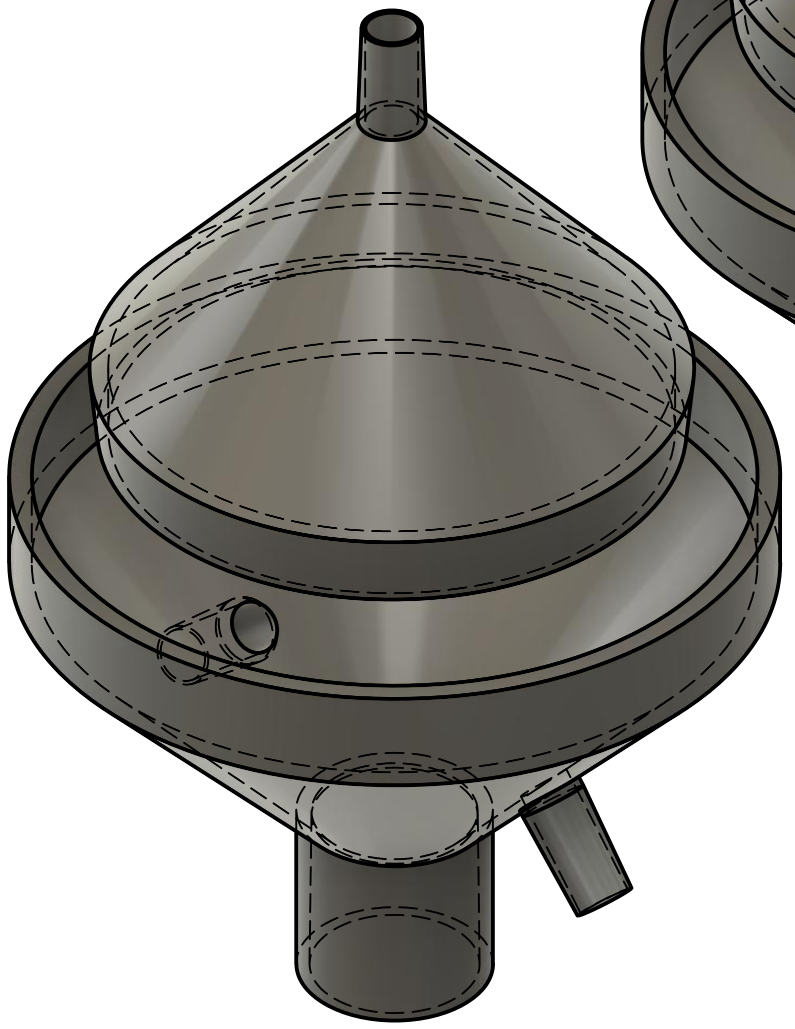

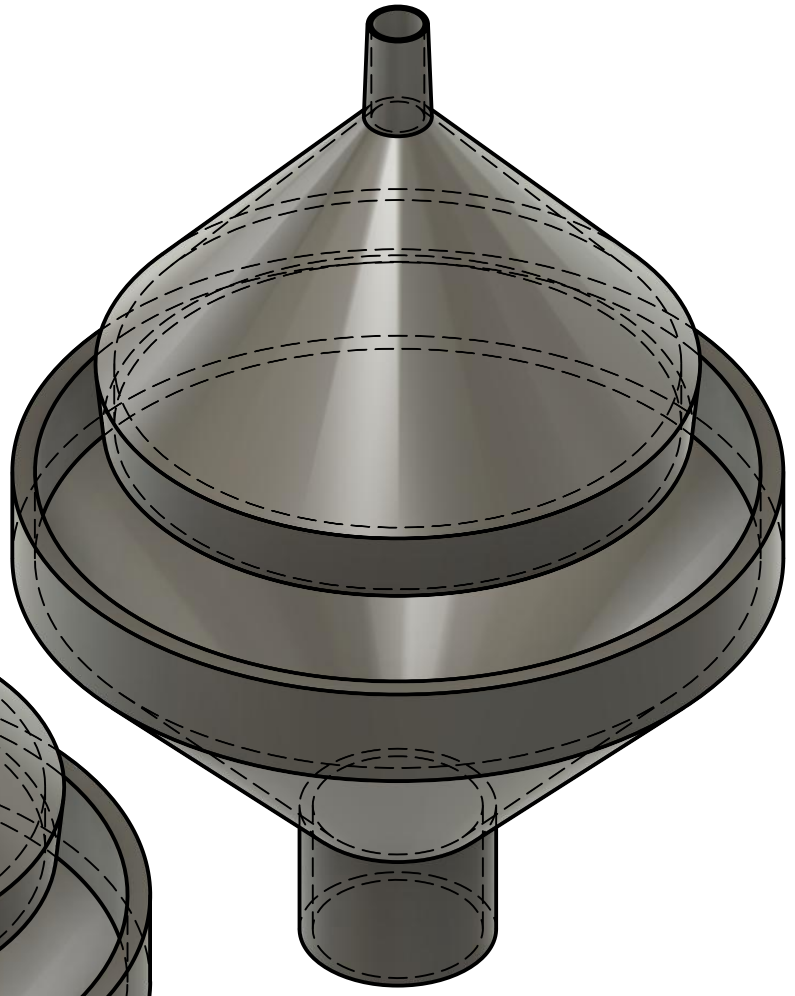

Dept.	Technical reference	Created by	Approved by
		Nicholas Markin 1/11/22	
		Document type	Document status
		Title: COVID Evacuator adapter	DWG No.
			Rev. / Date of issue / Sheet 1/1
