# Supplementary figures and images for: Infectious Aerosol Capture Mask as Environmental Control to Reduce Spread of Respiratory Viral Particles
Source: Viruses. 2022 Jun 11;14(6):1275. doi: 10.3390/v14061275 (PMC9227466; doi:10.3390/v14061275)

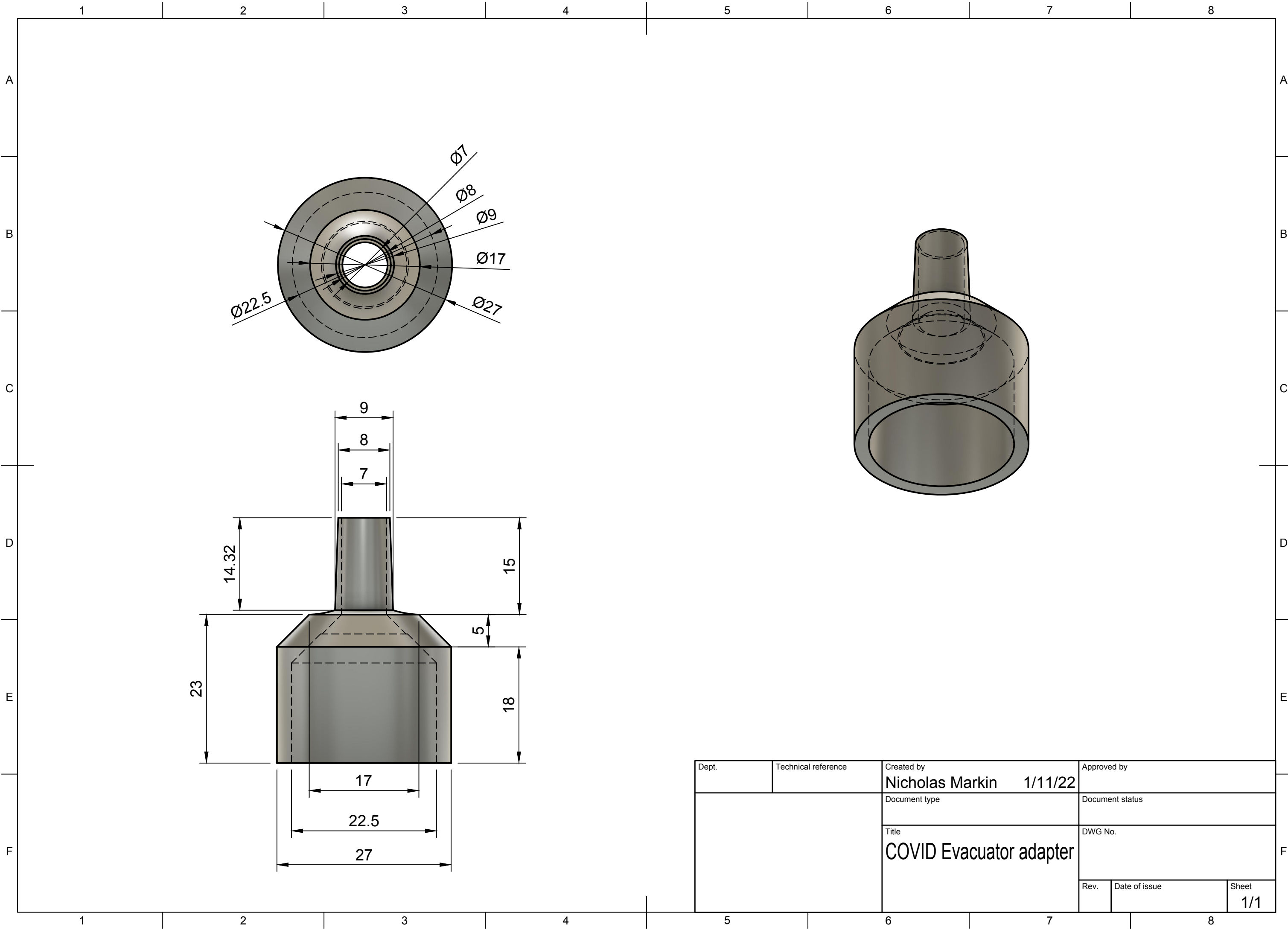

Supplement: Supplementary file 1 [file viruses-14-01275-s001.zip › Figure S1.pdf]

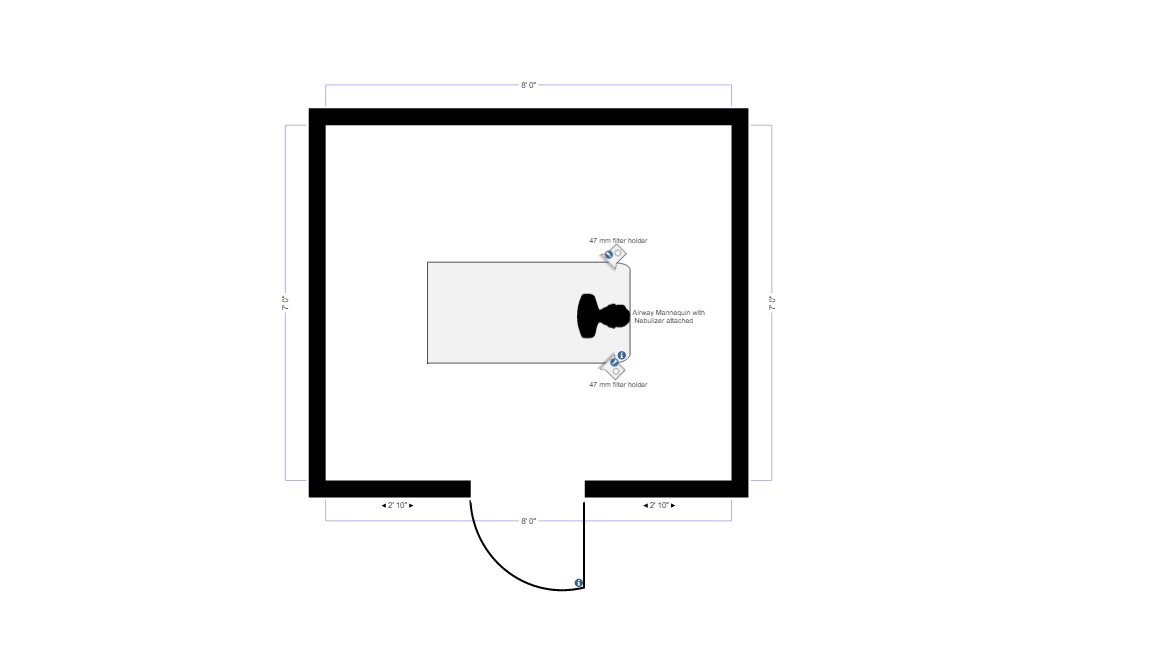

Supplement: Supplementary file 1 [file viruses-14-01275-s001.zip › Figure S3.jpg]
